# Rampant historical mitochondrial genome introgression between two species of green pond frogs, *Pelophylax nigromaculatus *and *P. plancyi*

**DOI:** 10.1186/1471-2148-10-201

**Published:** 2010-06-29

**Authors:** Kui Liu, Fang Wang, Wei Chen, Lihong Tu, Mi-Sook Min, Ke Bi, Jinzhong Fu

**Affiliations:** 1 College of Life Sciences, Capital Normal University, Beijing 100048, China; 2 College of Veterinary Medicine, Seoul National University, Seoul 151-742, South Korea; 3 Department of Integrative Biology, University of Guelph, Guelph, Ontario N1G 2W1, Canada

## Abstract

**Background:**

Mitochondrial introgression may result in the mitochondrial genome of one species being replaced by that of another species without leaving any trace of past hybridization in its nuclear genome. Such introgression can confuse the species genealogy estimates and lead to absurd inferences of species history. We used a phylogenetic approach to explore the potential mitochondrial genome introgression event(s) between two closely related green pond frog species, *Pelophylax nigromaculatus *and *P. plancyi*.

**Results:**

DNA sequence data of one mitochondrial and two nuclear genes from an extensive sampling of the two species were collected, and the genealogies of the three genes were constructed and compared. While the two nuclear genes congruently showed mutual reciprocal monophyly of both species, the mitochondrial phylogeny separated a Korean *P. nigromaculatus *clade, a paraphyletic central China *P. plancyi *assemblage, and a large well-supported introgression clade. Within the introgression clade, the mitochondrial haplotypes of the two species *were *mixed together. This reticulated pattern can be most parsimoniously explained by an ancient mitochondrial introgression event from *P. plancyi *to *P. nigromaculatus *that occurred at least 1.36 MYA, followed by multiple recent introgression events from *P. nigromaculatus *back to *P. plancyi *within the last 0.63 MY. The re-constitution of previously co-adapted genomes in *P. plancyi *may be responsible for the recent rampant introgression events. The Korean *P. nigromaculatus *clade likely represents the only surviving "true" mitochondrial lineage of *P. nigromaculatus*, and the central China *P. plancyi *assemblage likely represents the "original" *P. plancyi *mitochondrial lineage. Refugia in the Korean Peninsula and central China may have played a significant role in preserving these ancient lineages.

**Conclusions:**

The majority of individuals in the two species have either introgressed (*P. nigromaculatus*) or reclaimed (*P. plancyi*) mitochondrial genomes while no trace of past hybridization in their nuclear genomes was detected. Asymmetrical reproductive ability of hybrids and continuous backcrossing are likely responsible for the observed mitochondrial introgression. This case is unique in that it includes an ancient "forward" introgression and many recent "backward" introgressions, which re-constitutes the original nuclear and mitochondrial genomes of *P. plancyi*. This hybrid system provides an excellent opportunity to study cyto-nuclear interaction and co-adaptation.

## Background

Historical mitochondrial introgression often results in the mitochondrial genome of one species being replaced by that of another species without leaving any trace of hybridization in its nuclear genome. The introgressed genome can become fixed in some populations and extend to a large portion of the recipient species' distribution. Wilson and Bernatchez termed this phenomenon "ghost of hybrids past" [1]. For example, Melo-Ferreira et al. found mitochondrial introgression from the mountain hare (*Lepus timidus*) to three other hare species, *L. granatensis*, *L. europaeus *and *L. castroviejoi *in the Iberian Peninsula [2,3]. Although *L. timidus *is currently not sympatric with the three Iberian hares, distribution range expansion and retraction during glaciation might have created opportunities for the species to hybridize. Selective advantage of the *L. timidus *mitochondrial genome over others was attributed to the widespread presence of the introgressed mitochondrial genome. McGuire et al. also presented a case study in lizards of the genus *Crotaphytus *[4]. The mitochondrial genome of *C. collaris *has replaced that of *C. reticulatus *in approximately two-thirds of its range *via *an ongoing selective sweep. A unidirectional mitochondrial introgression also took place from *C. collaris *to *C. bicinctores*. Introgressive hybridization may have occurred repeatedly but was temporally separated throughout at least the latter half of the Pleistocene. Other cases of mitochondrial introgression have been reported in diverse metazoan taxa including carabid beetles [5], fruit flies [6], brook charr [7], megophryid frogs [8], Sika deer [9], African elephants [10], and pocket gophers [11].

Such processes have a number of significant implications in biology. (1) Mitochondrial gene introgression can confuse the estimated genealogy of a species, because an introgressed genome will not reveal any history before the introgression events [12] and the mix of introgressed and original genomes within a species could lead to absurd inferences of the species history. Therefore, cautions should always be exercised when using mitochondrial DNA (mtDNA) alone to infer demography and evolutionary history of a species. This is important because in the last three decades, we have witnessed a great number of "phylogeographic" studies using mtDNA as a sole marker [13,14]. (2) Introgression often disrupts the co-evolution between the mitochondrial and nuclear genomes. Genes from both genomes encode several proteins that are critical to metabolism and an abrupt disassociation between the genomes may interfere not only their respective normal function but also the established genomic interactions and co-adaptation [15]. (3) Massive introgression events were often suggested to be adaptive processes driven by natural selection [1,4,12]. The spontaneous breakdown and reconstruction of mitochondrial-nuclear genome associations may open a unique window to examine the adaptation process of a newly introgressed mitochondrial genome, as well as to better understand the interactive dynamics and co-adaptive functioning between mitochondrial and nuclear genomes.

**Figure 1:**
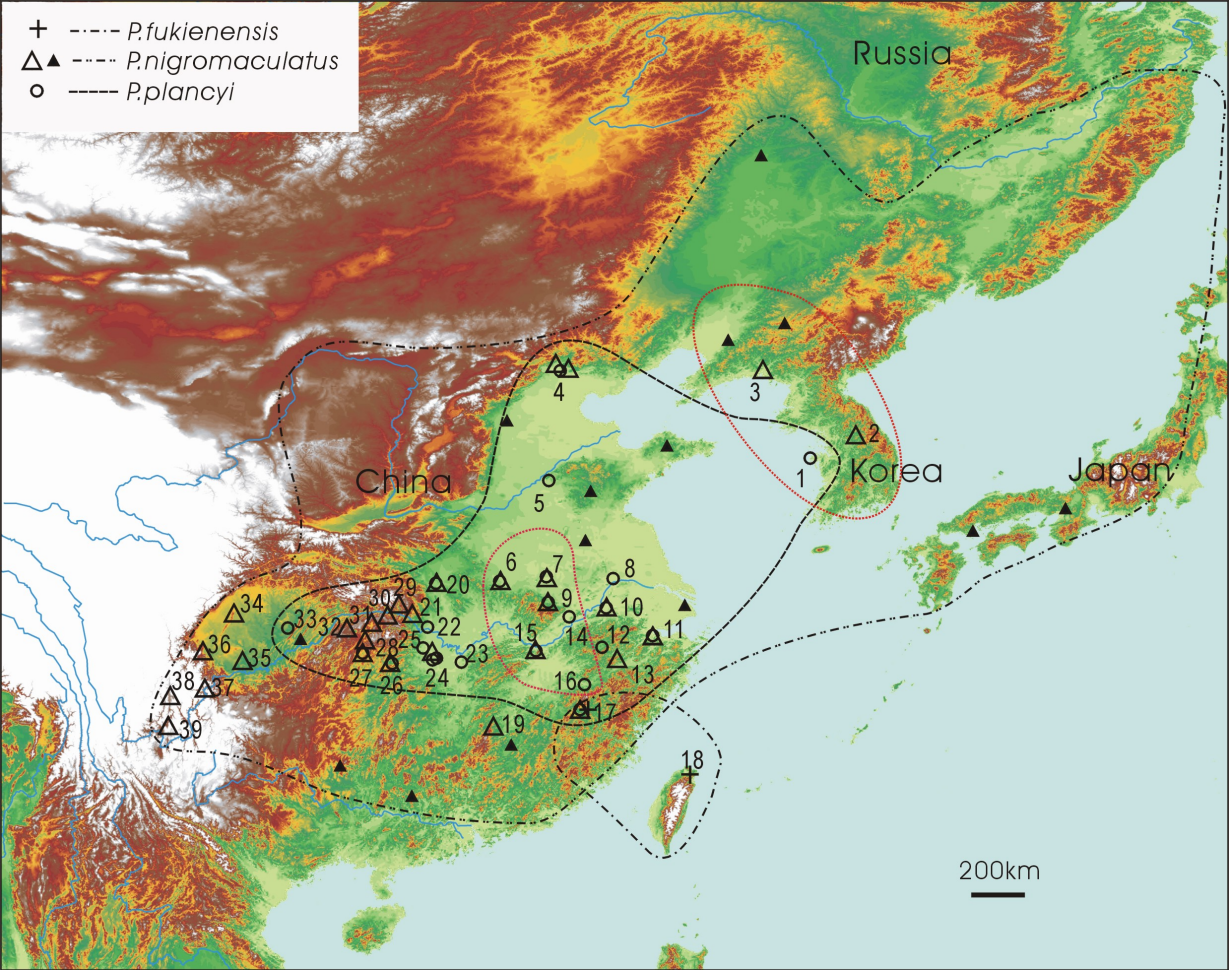
**Species distribution map and sampling sites of *Pelophylax fukienensis, P. nigromaculatus *and *P. plancyi***. Cross = *Pelophylax fukienensis*; Circle = *P. plancyi*; Triangle = *P. nigromaculatus*. Solid triangles represent samples from GenBank. Only selected sampling sites from GenBank are presented here to demonstrate geographic coverage. The red dotted lines outline the distribution of two ancient lineages of "original" mitochondrial genomes, which are possibly associated with the assumptive Korean refugium and the central China refugium during glaciations.

Kim et al. first suggested a possible mitochondrial genome introgression between two green pond frog species, *Pelophylax nigromaculatus *and *P. plancyi chosenicus *[16]. Using mitochondrial cytochrome *b *partial sequences, they found that the Japanese *P. nigromaculatus *is more closely related to Korean *P. plancyi chosenicus *than to Korean *P. nigromaculatus*, while their nuclear gene data (allozymes) unequivocally group the *P. nigromaculatus *populations together. Subsequently, they hypothesized a possible mitochondrial genome introgression event between the two species. Nevertheless, the extent, timing and directions of introgression were unresolved because of their small sample size (n = 7). Green pond frogs in the genus *Pelophylax *(formerly part of the genus *Rana*) are no stranger to inter-specific hybridization. *Pelophylax esculentus*, a species of hybrid origin between *P. lessonae *and *P. ridibunda*, is probably the best studied case of inter-specific hybridization [17]. Both *P. nigromaculatus *and *P. plancyi *are common and widespread in eastern Asia, and their distribution ranges largely overlap (Figure 1). Ecologically, *P. nigromaculatus *is a generalist and occurs in a wide range of habitats including small to large ponds and rivers. On the other hand, *P. plancyi *requires more specific habitats and is most commonly seen in large well-vegetated ponds, particularly those with lotus plants. Both species are spring breeders and occasionally use the same breeding sites. The gross morphology of the two species is similar but they can be easily distinguished. While males of *P. nigromaculatus *have a pair of external vocal sacs, males of *P. plancyi *have a pair of internal ones or do not have vocal sacs at all. In addition, *P. plancyi *has a conspicuous dark line along the back of its thigh that is lacking in *P. nigromaculatus*. Several other names have been proposed for geographic populations of *P. plancyi*, including *P. fukienensis, P. hubeiensis*, and *P. chosenicus*. Some authors treat them as valid species [18] while others treat them as subspecies of *P. plancyi *([19]; see Frost [20] for references and comments). In this study, we refer all populations allocated to these names as the *P. plancyi *complex to facilitate our presentation.

Following the lead of Kim et al.'s study [16], we use a phylogenetic approach to explore the potential mitochondrial genome introgression between *P. nigromaculatus *and *P. plancyi*, and to detail its extension in both time and space by extensive sampling of both species across their ranges. Recent development in DNA sequencing technology allows us to obtain large amounts of sequence data from both the mitochondrial and nuclear genomes. By comparing their gene genealogies, gene introgression events can be revealed. If there was no historical mitochondrial introgression between *P. nigromaculatus *and *P. plancyi*, both the nuclear and mitochondrial genes would group haplotypes from each of the two species respectively in the same fashion, and Kim *et al*.'s hypothesis [16] would be rejected. If introgressions occurred between the two species, a phylogeny based on nuclear genes would group haplotypes from each of the two species, but part or all of the mitochondrial haplotypes from one species would nest within the other species.

## Results

### Nuclear proopiomelanocortin gene *(POMC) *and tyrosinase gene *(TYR) *genealogies

Sequence data were collected from two nuclear genes (*POMC *and *TYR*). We first conducted a recombination test for the nuclear gene data and did not detect any recombination event at both global and pairwise levels. Therefore, all data were proceeded for phylogenetic analyses without modification. Both Bayesian inference and maximum parsimony methods were used to construct the gene genealogy.

A total of 66 *POMC *sequences from 59 samples for *Pelophylax nigromaculatus *(including one from GenBank) and 488 sequences from 321 samples for *P. plancyi *complex were gathered. Two sequences for two outgroup taxa, *Lithobates catesbeianus *and *Staurois latopalmatus *were gathered from GenBank. A total of 611 nucleotide sites were confidently resolved and 139 haplotypes were identified, including two for the outgroup taxa. Among the ingroup members, 83 sites were variable.

**Figure 2:**
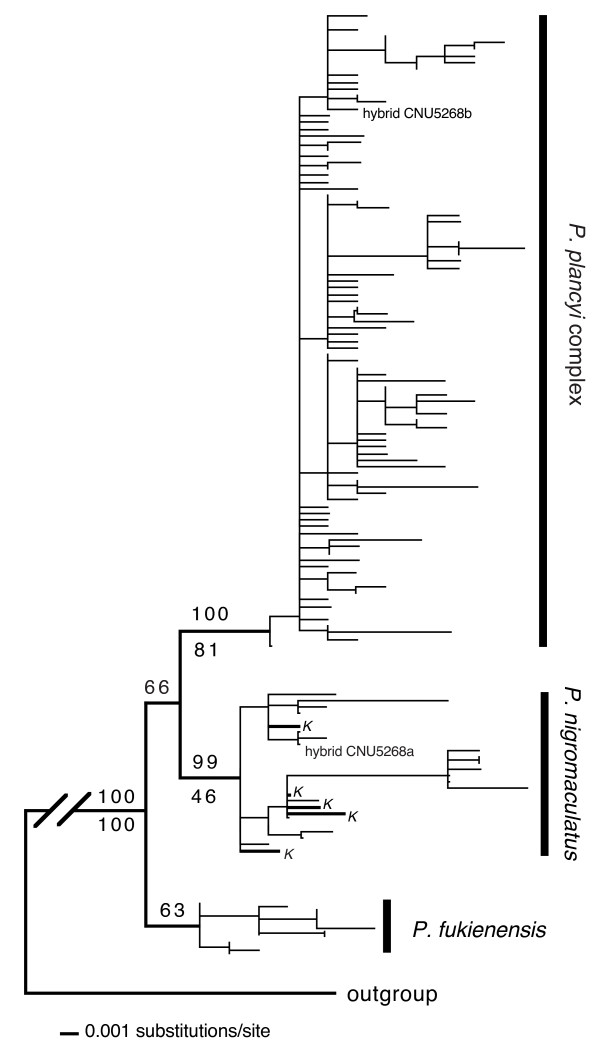
**A Bayesian tree from the *POMC *gene data**. The numbers above the branches are Bayesian posterior probabilities and the numbers below the branches are bootstrap proportions from the parsimony analysis. Bold lines and the "*K*" indicate haplotypes from the Korean *nigromaculatus *clade

For the Bayesian analysis, the HKY+I+G model was selected as the best-fit model by the hierarchical likelihood ratio test. Figure 2 shows one of the Bayesian trees with posterior probabilities of the basal nodes mapped on the tree. Both the resolution of the Bayesian consensus tree and the posterior probabilities were low (Figure 2); this was not surprising given the small number of variable sites. However, three clades were clearly depicted on the tree with moderate to high nodal supports. A *P. plancyi *clade included all haplotypes from *P. plancyi *complex except *P. fukienensis*, and a *P. nigromaculatus *clade included all haplotypes of *P. nigromaculatus *except CNU5268. CNU5268 (site 15) was a heterozygote and its two haplotypes were grouped in the *P. plancyi *clade and the *P. nigromaculatus *clade, respectively. Both clades received high posterior probabilities (0.99 and 1.00). A third clade, the *P. fukienensis *clade, included all samples from *P. fukienensis*, with a low posterior probability (0.63). For the maximum parsimony analysis, 41 sites were phylogenetically informative. The parsimony analysis found more than 400,000 equally most parsimonious trees (MPTs) with 130 steps, a consistency index (CI) of 0.4538 and a retention index (RI) of 0.8483. The strict consensus tree (tree not shown) was similar but less resolved compared to the Bayesian consensus tree. Again, a *P. nigromaculatus *clade and a *P. plancyi *clade were resolved with exactly the same membership as the Bayesian tree. The relationships within each clade were largely unresolved. However, the haplotypes of *P. fukienensis *samples did not form a monophyletic group. Rather, they formed a paraphyletic assemblage at the base of the tree.

A total of 77 *TYR *sequences from 60 samples for *Pelophylax nigromaculatus *(including three from GenBank) and 481 sequences from 321 samples for *P. plancyi *were gathered. Three sequences for three outgroup taxa, *Babina pleuraden*, *Rana shuchinae*, and *Pelophylax lessonae*, were gathered from GenBank. A total of 601 nucleotide sites were confidently resolved and 79 haplotypes were identified, including three for the outgroup. Among the ingroup members, 42 sites were variable and 23 were phylogenetically informative.

**Figure 3:**
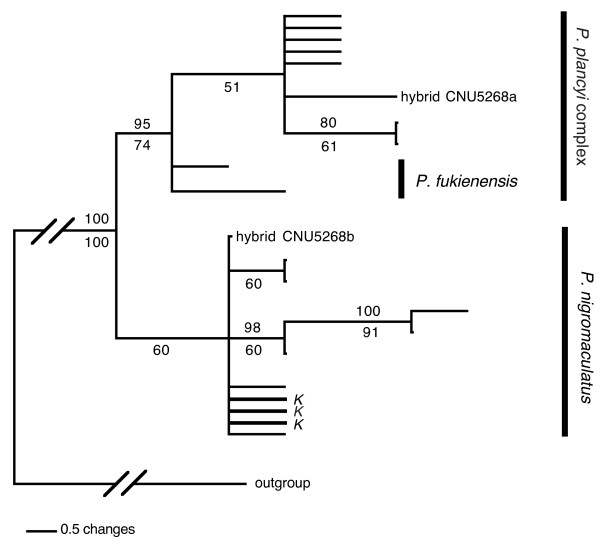
**The simplified phylogram of the strict consensus tree from the parsimony analysis of the *TYR *gene data**. The numbers above the branches are Bayesian posterior probabilities from the Bayesian analysis and the numbers below the branches are bootstrap proportions. Bold lines and the "*K*" indicate haplotypes from the Korean *nigromaculatus *clade; all these haplotypes are shared between members of the Korean *nigromaculatus *clade and others.

The parsimony analysis found better-resolved trees than did the Bayesian analysis, and therefore, a simplified phylogram of the strict consensus tree from the parsimony analysis is presented in Figure 3. The analysis found more than 400,000 MPTs with 93 steps (CI = 0.5161; RI = 0.8193). Both the resolution of the tree and the bootstrap supports were low, which was not surprising given the small number of informative characters. However, two clades, a *P. nigromaculatus *clade and a *P. plancyi *clade (including *P. fukienensis*) were clearly depicted on the strict consensus tree with moderate nodal supports (Figure 3). For the Bayesian analysis, a SYM+I+G model was selected as the best-fit model by the hierarchical likelihood ratio test. Similar to the parsimony tree, the Bayesian tree also had low resolution and nodal supports (tree no shown). All haplotypes of the *P. plancyi *complex, including *P. p. fukienensis*, were grouped together, in the same way as in the parsimony tree, with a posterior probability of 0.96. However, the haplotypes of *P. nigromaculatus *did not form a clade, rather they formed a paraphyletic assemblage at the base of the tree.

Both *POMC *and *TYR *genes produced two well-defined clades: a *P. nigromaculatus *clade that includes all haplotypes of the *P. nigromaculatus *samples excluding CNU5268 and a *P. plancyi *clade that included all haplotypes of the *P. plancyi *complex excluding haplotypes of *P. fukienensis*. Both clades received moderate to high supports from both genes. The *TYR *gene tree had a lower resolution than the *POMC *gene tree; although the Bayesian analysis of *TYR *gene failed to group all haplotypes of *P. nigromaculatus *together (parsimony analysis did), members of the two clades were always well separated on every tree. A third group, the *P. fukienensis *clade that included all samples of *P. fukienensis*, was weakly supported by the *POMC *gene data, and not supported (neither contradicted) by the *TYR *gene data. Examining the sequence data directly, we found several fixed differences between the three clades. There were 2, 2, and 1 sites of the *POMC *gene and 2, 1, and 0 sites of the *TYR *gene that are fixed for the *P. nigromaculatus *clade, the *P. plancyi *clade and the *P. fukienensis *clade, respectively.

One individual (CNU5268) appeared to be an F_1 _hybrid between *P. plancyi *and *P. nigromaculatus*. Two haplotypes from this individual were obtained by cloning and were grouped with the *P. plancyi *clade and the *P. nigromaculatus *clade, respectively. Directly examining the sequences, we found that CNU5268 was heterozygous at all polymorphic sites between *P. plancyi *and *P. nigromaculatus*. For example, at position 90 of *POMC *gene, all *P. plancyi *samples had a "C" while all *P. nigromaculatus *samples had a "T". CNU5268 was heterozygous on the site with "C" and "T". However, we cannot eliminate the possibility that this individual resulted from a recent backcross, e.g. F _2 _or F _3_, because we only examined two gene loci.

### Mitochondrial cytochrome b gene *(Cyt-b) *genealogy

**Figure 4:**
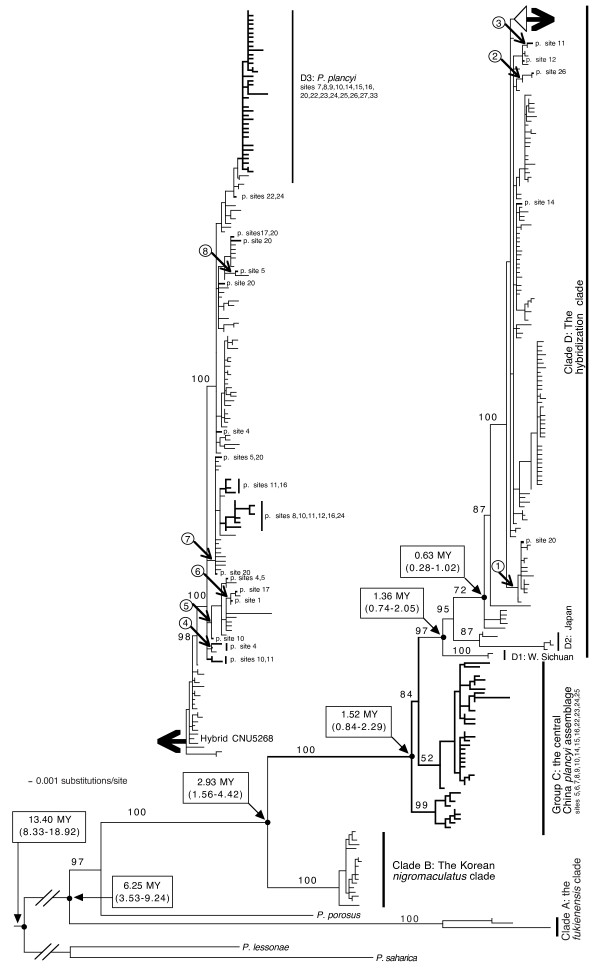
**A Bayesian tree from the mitochondrial cytochrome *b *gene data**. Bold branches and p. represent haplotypes of *Pelophylax plancyi *and others are *P. nigromaculatus *unless otherwise indicated. The numbers above the branches are Bayesian posterior probabilities. Divergence times were estimated from a reduced data set; divergence times in parentheses are confidence intervals at 95% level. Numbers within circles indicated close association between haplotypes of *P. nigromaculatus *and *P. plancyi*, which likely represent independent mitochondrial genome introgression events.

Sequence data were collected from one mitochondrial gene, *Cyt-b*. A total of 335 *Cyt-b *sequences were gathered for the *P. plancyi *complex, including 333 new sequences from this study and two sequences from GenBank. A total of 330 *Cyt-b *sequences were gathered for *P. nigromaculatus*, including 57 new sequences from this study and 273 sequences from several published sources. In addition, four sequences for three outgroup taxa, *P. saharicus*, *P. lessonae*, and *P. porosus *were obtained from GenBank. Sequences from different studies had different lengths; approximately half of the sequences included 1,043 nucleotide sites (full length) while others has 670-850 sites at various positions. To accommodate the majority, a data set of 1043 sites was created. A total of 380 haplotypes were identified, including three for the outgroup taxa, and 393 sites were variable and 269 were phylogenetically informative. A GTR+I+G model was selected as the best-fit model by the hierarchical likelihood ratio test. A Bayesian tree of *Cyt-b *along with the posterior probabilities is provided in Figure 4.

The Bayesian tree revealed several interesting features (Figure 4). Similar to the *POMC *gene trees (Figure 2), it resolved a *P. fukienensis *clade (clade A) at the very base of the tree. The member composition of the clade was exactly the same as the *POMC *tree. One outgroup taxon, *P. porosus*, was placed between the *P. fukienensis *clade and the other ingroup members. Unlike the nuclear gene trees, haplotypes of *P. nigromaculatus *and *P. plancyi *did not form two monophyletic groups, rather, they were intermingled and together formed one monophyletic group. Within this *plancyi-nigromaculatus *group, a Korean *P. nigromaculatus *clade (clade B) branched off first, which included all the *P. nigromaculatus *samples from the Korean Peninsula and adjacent locations of China (sites 2 and 3 and surrounding area; Figure 1). The sistergroup of the Korean *P. nigromaculatus *clade included a paraphyletic assemblage (group C) of haplotypes of *P. plancyi *from central China (sites 5, 6, 7, 8, 9, 10, 12, 14, 15, 16, 22, 23, 24 and 25 but predominantly sites 6, 7, 9, 15, and 16; Figure 1) at the base and a well-supported monophyletic group (clade D: the introgression clade) nested inside the paraphyletic group C. Clade D was the most interesting group and included the remaining *P. nigromaculatus *and *P. plancyi *haplotypes. Within this clade, again, haplotypes from *P. nigromaculatus *and *P. plancyi *failed to form respective clades; haplotypes of *P. plancyi *sporadically nested within "bushes" of *P. nigromaculatus *at 20 different places, except at the top of the tree where a large number of haplotypes of *P. plancyi *formed a clade (D3). The basal branches of clade D comprised haplotypes of *P. nigromaculatus*. Two haplotypes of *P. nigromaculatus *from western Sichuan and five haplotypes from Japan formed two well-supported groups (D1 and D2) at the very basal position of clade D. Although the overall nodal supports within clade D were low, some close associations between haplotypes of *P. nigromaculatus *and *P. plancyi *were well supported. Eight clades, where haplotypes from both species were found, received BPPs greater than 0.90 (Figure 4). Within each of these eight clades, haplotypes of *P. nigromaculatus *and *P. plancyi *were found to be identical or only different by one or two base pairs. In addition, the overall clado-pattern of the haplotypes within clade D bore little association with their geographic locations, except a few well-supported sister groups in which both members came from the same area.

The parsimony analysis found more than 400,000 MPTs with 879 steps (CI = 0.3936, RI = 0.8733). Similar to the Bayesian tree, the MP tree also defined a *P. fukienensis *clade, clade (B+C+D), clade B, clade (C+D), a paraphyletic group C, and several other nodes within clade D. Several close associations between *P. nigromaculatus *and *P. plancyi *within clade D were also identified. However, the other relationships among the haplotypes were poorly resolved compared to the Bayesian tree.

The estimated times of divergences between the major mitochondrial lineages were mapped on the *Cyt-b *gene tree (Figure 4). In general, these estimates were largely congruent with several other independent studies. For example, Lymberakis et al. estimated that the European and Far Eastern lineages were separated 15 MYA [21], and Sumida *et al*.'s estimate for this dichotomy was 5.9-10.9 MYA based on allozyme data [22]. These results were relatively close to our estimate of 13.40 MYA (CI 8.33-18.92). Sumida et al. estimated that the split of *P. nigromaculatus *and *P. porosus *took place at least 3 MYA [23], and it was again congruent with our estimate of 2.93 to 6.25 MYA for this event.

There were major differences between the nuclear and mitochondrial gene trees. Both nuclear genes resolved both *P. plancyi *and *P. nigromaculatus *as monophyletic clades, but the mitochondrial gene tree mixed haplotypes from the two species together. On the other hand, a major agreement among the nuclear (*POMC*) and mitochondrial gene trees was that all of them resolved a separated *P. fukienensis *clade. All new sequences are deposited in GenBank (accession numbers: *Cyt-b *GU977277-GU977669; *POMC *GU977670-GU978222; *TYR *GU978223-GU978775). Aligned data sets that used to generate the phylogenetic hypotheses are presented as additional files 1, 2 and 3.

## Discussion

### The multiple mitochondrial genome introgression hypothesis

The haplotypes of *P. nigromaculatus *and *P. plancyi *intertwined together on the mitochondrial gene tree (Figure 4), despite the clear separation between the two species on both nuclear gene trees (Figure 2 and Figure 3). Other evidence, such as morphological and ecological traits, also strongly supports the validity of the two species [18]. The conflicting patterns between the nuclear and mitochondrial gene trees support Kim *et al*.'s hypothesis [16] that there were historical mitochondrial introgressions between *P. nigromaculatus *and *P. plancyi. *To best explain our data, we further propose a multiple mitochondrial genome introgression hypothesis: one ancient "forward" mitochondrial introgression event from *P. plancyi *to *P. nigromaculatus *followed by rampant recent "backward" introgression events from *P. nigromaculatus *to *P. plancyi*.

Our nuclear gene data conform to several previous studies and suggest that the *P. plancyi *and *P. nigromaculatus *are sistergroups [18,21]. The Korean *P. nigromaculatus *clade (clade B) on the *Cyt-b *gene tree likely represents the only "true" mitochondrial lineage of *P. nigromaculatus*, and its sistergroup (clade C+D) likely represents the mitochondrial lineage of *P. plancyi *(Figure 4). The paraphyletic group C (the central China *P. plancyi *assemblage) likely represents the "original" mitochondrial lineages of *P. plancyi*, which have always been associated with *P. plancyi *nuclear genomes. The introgression clade D, which nests within group C, represents an introgressed *P. plancyi *mitochondrial lineage (Figure 4). Within this clade, *P. plancyi *individuals are sporadically dispersed throughout the "bushes" of *P. nigromaculatus*, and the four basal sub-clades comprise *P. nigromaculatus *individuals exclusively. The most parsimonious explanation for this pattern is that all individuals in this clade share a common mitochondrial ancestor derived from an ancient hybridization event between a female *P. plancyi *and a male *P. nigromaculatus*. The introgressed *P. plancyi *genome subsequently expanded to the majority of the distribution range of *P. nigromaculatus *except the Korean Peninsula and its adjacent areas. The nested *P. plancyi *lineages represent *P. plancyi *individuals, which "took back" their mitochondrial genomes from *P. nigromaculatus via *recent hybridization events. Of the 20 nested placements of *P. plancyi*, there are cases where the closest *P. nigromaculatus *relatives are not identified due to the unresolved relationships on the tree. Nevertheless, there are eight well-supported sub-clades (BPP >0.90) that embrace both *P. plancyi *and *P. nigromaculatus *individuals. Each of these sub-clades likely represents a recent mitochondrial introgression event from *P. nigromaculatus *to *P. plancyi*. The introgressions between the two species appear to be bi-directional and, at present, the majority of both species possess introgressed, rather than "original", mitochondrial genomes.

Overall, the multiple mitochondrial genome introgression hypothesis most parsimoniously accounts for our data and also immediately provides an explanation for the rampant introgression within the introgression clade (clade D). In this clade, all *P. nigromaculatus *individuals do, in fact, possess historical mitochondrial genomes of *P. plancyi*, and the mitochondrial introgression from *P. nigromaculatus *to *P. plancyi *actually results in re-union of the mitochondrial and nuclear genomes of *P. plancyi*. The two genomes are already co-adapted, and therefore, the introgression might have little negative impact on the fitness of the hybrids.

A re-examination of the mitochondrial gene tree of the two species, by increasing the number of taxa or number of informative characters (nucleotide sites), may change the topology and favor a different explanation. However, our sampling of taxa (populations) is robust and more taxa are unlikely to change the topology. More data could improve the resolution of the tree, particularly within clade D. Those will unlikely change the essentials of the current hypothesis but will likely help to resolve the number of recent introgression events within clade D. The intertwined pattern of the mitochondrial gene tree may also be explained by incomplete lineage sorting [12], however, it is probably not valid in our case. Due to the maternal mode of inheritance, mitochondrial genes are expected to reach coalescence up to four times faster than nuclear genes [24]. In our study, both nuclear genes (particularly *POMC*) have reached coalescence at the species level, and it is therefore unlikely that the more rapidly evolving mitochondrial genes have not.

### Diversification and introgression of the *P. nigromaculatus-plancyi *mitochondrial genomes in time and space

The Korean *nigromaculatus *clade (clade B), which includes all samples from the Korean Peninsula and adjacent locations of China, was separated from the rest at approximately 2.93 MYA (Figure 4). Since the Korean *nigromaculatus *clade represents the only "true" *P. nigromaculatus *mitochondrial lineage while all other *P. nigromaculatus *and *P. plancyi *possess a *P. plancyi *mitochondrial genome, this split may coincide with the speciation event that separated *P. nigromaculatus *and *P. plancyi*. All other *P. nigromaculatus *possess an introgressed *P. plancyi *mitochondrial genome. This "true" *P. nigromaculatus *mitochondrial lineage might have survived in the Korean Peninsula refugium during ice ages and expanded to nearby areas of China during the inter-glaciation periods (sites 2 and 3, Figure 1). The Korean Peninsula is a well-recognized refugium during several glaciation cycles and has provided shelters for many ancient lineages [25,26]. A recent phylogeographic study of *P. nigromaculatus *by Zhang et al. also identified two major clades, a Korean clade including samples from the Korean Peninsula and adjacent China and a main clade including the Japanese and most Chinese samples [27]. Without realizing that the main clade of *P. nigromaculatus *was actually descended from *P. plancyi*, they hypothesized that the Gunz glaciation (0.9-1.2 MYA) might be the cause of allopatric isolation and lineage splitting. Our divergence time estimate (2.93 MYA) is older than that of Zhang *et al*.'s [27], and the observed divergence is not a lineage split within *P. nigromaculatus*, rather, it represents the separation between the two species.

The central China *P. plancyi *mitochondrial lineages share a most recent common ancestor with clade D at approximately 1.52 MYA (Figure 4). The central China lineages may represent the only survivors of the "original" *P. plancyi *mitochondrial genome. The majority members of these lineages are from sites 6, 7, 9, 15 and 16 in the vicinity of Da-Bie-Shan Mountains (site 9; Figure 1). The persistence of these ancient lineages may be attributed to the Da-Bie-Shan refugium. The Da-Bie-Shan and surrounding area is hypothesized to have been a glaciation refugium since the Tertiary and maintains many ancient endemic species [28,29]. These *P. plancyi *mitochondrial lineages may have survived in the refugium and expanded to the surrounding area (sites 5, 6, 7, 8, 9, 10, 12, 14, 15, 16, 22, 23, 24, and 25; Figure 1).

Clade D represents an ancient mitochondrial genome introgression event that occurred at least 1.36 MYA (CI 0.74-2.05). Most previous reported cases of mitochondrial introgression are recent and the introgressed mitochondrial genomes in the recipient species are identical or nearly so to those in the donor species [2,6]. The two reported exceptions are crotaphytid lizards and *Scutiger *frogs. McGuire et al. reported repeated introgression events between *Crotaphytus collaris *and *C. bicinctores *and some could be as old as 2.5 MYA [4]. Chen et al. reported a case between two species of *Scutiger*, which was dated at approximately 3.5-9.5 MYA [8]. With increasing haplotype divergence, it becomes more difficult to discriminate between introgression and incomplete lineage sorting [30], but our data provide a convincing case of ancient mitochondrial genome introgression. We could not locate where this introgression event might have occurred from our data; repeated glaciation circles and associated range expansions/contractions may have rendered it difficult, if not impossible.

Within the introgression clade (D), multiple introgression events have taken place over time and space. There are at least eight well-supported sub-clades that demonstrate close associations between *P. nigromaculatus *and *P. plancyi *and represent at least eight independent introgression events. If we consider those sub-clades that did not receive high nodal supports, introgressions from *P. nigromaculatus *to *P. plancyi *may have independently taken place as many as 20 times. All these introgression events are probably recent. The common ancestor of the clade that includes most of clade D excluding D1 and D2, is 0.63 MYA (CI 0.28-1.02); therefore, all divergence within this clade should be younger than this time estimate. Within these eight well-supported sub-clades, the differences between *P. nigromaculatus *and *P. plancyi *are small: some are identical while the largest differences are two of the 670 base pairs that were compared. Furthermore, the introgression events may have occurred at various locations. Within the eight well-supported sub-clades, closely associated haplotypes of *P. nigromaculatus *and *P. plancyi *are often from the same area. For example, all individuals of both species in sub-clade 2 are from Zhang Jia Jie (site 26), and in sub-clade 3, all individuals are from Zhejiang Province of China (sites 11 and 13). Interestingly, the Korean *P. plancyi *is not closely related to the Korean *P. nigromaculatus*, suggesting that the Korean *P. plancyi *may not be indigenous, and is probably recently established. In addition, the hybridization between the two species appears to be an ongoing process and this is evidenced by an F1 hybrid identified in the present study.

### Mechanisms of mitochondrial genome introgression and replacement

Asymmetrical reproductive ability of hybrids and continuous backcrossing are likely responsible for the observed mitochondrial introgression and replacement between the two green pond frogs. Kawamura and Nishioka reported that all male F _1 _hybrids between *P. nigromaculatus *and *P. plancyi chosenicus *were sterile, but half of the female F _1 _hybrids were probably fertile [31]. Both field observation and lab experiments also confirmed that among crosses between *P. nigromaculatus *and *P. porosus*, females were partially fertile but males were completely sterile [32]. Haldane's rule predicts that when in F _1 _offspring of two animal species, one sex is sterile, the sex is usually the heterogametic sex. *Pelophylax nigromaculatus *has an XX/XY sex determination [33], and therefore, the above observations are consistent with Haldane's rule. While frequent inter-specific hybridization is the necessary first step toward mitochondrial genome introgression, such asymmetrical reproductive ability of hybrids would greatly facilitate backcrossing and the disappearance of hybridization signals in the nuclear genome while the introgressed mitochondrial genomes remain intact. Other mechanisms, such as asymmetries in species abundance or mating preferences and male-biased colonization [2,34] may also play a role in promoting mitochondrial introgression between the two green pond frogs.

The introgressed mitochondrial genomes in both *P*. *nigromaculatus *and *P. plancyi *expanded to the majority of their distribution ranges. McGuire et al. proposed two non-exclusive explanations for mitochondrial replacement across an extended geographic area: frequent hybridization events followed by genetic drift, or selective sweeps associated with rare hybridization [4]. The introgression from *P*. *nigromaculatus *back to *P. plancyi *is clearly a case of multiple rampant introgression events, and hence is consistent with McGuire et al's hypothesis [4]. On the other hand, the introgression from *P. plancyi *to *P*. *nigromaculatus *(the ancient introgression event) appeared to have occurred only once, so selective sweep might have been involved but we do not have data for this hypothesis. Thermal adaptation has been proposed for selection in several cases [7]. Nevertheless, no direct evaluation of the selective advantage of introgression event has so far been conducted. Theoretically, when species invade a new area, capturing an already adapted mitochondrial genome from the local species may provide the invader with a selective advantage [12].

### Taxonomic implications

Considering information from all three genes, *Pelophylax fukienensis *is a valid species. The nuclear gene *POMC *indicates that all its samples form a monophyletic group and possess fixed differences at several nucleotide sites from *P. plancyi*. At site 17, *P. fukienensis*, *P. nigromaculatus *and *P. plancyi *are sympatric. Of the 22 specimens that we examined, no hybrid was found, suggesting established reproductive isolation between the three species. Furthermore, the *P. fukienensis *clade is separated from all other *P. plancyi *by another valid species, *P. porosus*, on the mitochondrial gene tree. Pope first named the species, *"Rana" fukienensis *[35], but most recent authors considered it as a synonym of *P. plancyi *[19]. Based on molecular data, Sumida et al. recently suggested that it is a valid species, and is likely more closely related to *P. porosus *than to *P. nigromaculatus *[22]. Its distribution may include the island of Taiwan and the adjacent coast region of mainland China. However, many populations from inland of China, which were previously diagnosed as *P. fukienensis *(e.g. sites 10, 11, 12, 16 in [18]), are in fact *P. plancyi*.

Our data do not support the validity of *P. hubeiensis *and *P. chosenicus*. Both mitochondrial and nuclear genes failed to reveal any distinctiveness of the populations under the two names. The Korean populations of *P. plancyi *(=*P. chosenicus*) may have only invaded the Peninsula recently. Their reported morphological differences [18] are likely geographic intra-specific variations.

## Conclusions

There are multiple mitochondrial introgression events between the two green pond frog species, including one ancient "forward" introgression event from *P. plancyi *to *P. nigromaculatus *followed by rampant recent "backward" introgression events from *P. nigromaculatus *to *P. plancyi*. The majority of individuals in the two species have either introgressed (*P. nigromaculatus*) or reclaimed (*P. plancyi*) mitochondrial genomes while no trace of past hybridization in their nuclear genomes was detected. The mitochondrial introgression between these two species is unique in several ways. First, one introgression event is ancient, estimated at 1.36 MYA. Most previous reported cases of mitochondrial introgression are recent [2,6]. Second, independent introgression events from *P. nigromaculatus *to *P. plancyi *result in re-constitution of cyto-nuclear association of *P. plancyi*. This is the first such case. Third, there are multiple (as many as 20) but spatially and temporally separated introgression events. Such introgression is likely an ongoing process; the F _1 _hybrid that we found suggests that hybridization between the two species may frequently occur.

Mitochondrial genome introgression and replacement may be more common than we previously perceived. For example, inter-specific hybridization among amphibians is common, and mechanism that can produce mitochondrial introgression, such as asymmetrical reproductive ability of hybrids, is also commonly observed ([36,37]; Additional examples in Duellman and Trueb [38]). The common occurrence of such hybridization events likely has produced numerous mitochondrial genome introgressions in nature populations waiting to be detected.

Using mitochondrial genes alone to infer species history or to determine species status can be misleading, and mtDNA only represents an incomplete history of a species [12]. This message has been repeatedly demonstrated by numerous studies. A large number of mtDNA based phylogeographic studies and their conclusions may need to be revisited.

This mitochondrial introgression between *P. nigromaculatus *and *P. plancyi *provides a unique opportunity to study cyto-nuclear interaction and co-adaptation. Within *P. nigromaculatus *and *P. plancyi*, there are nuclear genomes interacting with their "original" and "co-evolved" mitochondrial genomes, and those interacting with "introgressed" foreign mitochondrial genomes. If the two genomes in every species were co-adapted, a recent mitochondrial introgression would most likely create mismatches between the mitochondrial vs. nuclear-encoded components (e.g. subunits for the electron transport chains) and cause functional disruption [15]. Such matches or mismatches can be examined and compared for different populations. Recent development in proteomics have provided us powerful tools to model the protein structure [39] and investigate the potential impact of these mismatches on the function of the protein molecules, which in turn may have significant impacts on the fitness of the hybrid lineages.

## Methods

### Sampling

An extensive coverage, particularly of the mitochondrial lineages of the two species, is essential for investigating the extent of mitochondrial introgression. A total of 333 specimens of the *Pelophylax plancyi *complex from 23 collecting sites were examined in this study, which included all four subspecies (*plancyi*, *fukienensis, hubeiensis*, and *chosenicus)*, and covered most of the species' distribution (Figure 1). In addition, mitochondrial *Cyt*-*b *gene sequences of one sample from the Korean Peninsula (AF205087) and one from the island of Taiwan (AB029941) were obtained from GenBank.

A total of 60 specimens of *P. nigromaculatus *from 29 collecting sites were included in this study, which covered the majority of the species' distribution and represented the three main lineages defined by a previous study [27]. In addition, 273 mitochondrial *Cyt-b *gene sequences representing samples from 75 locations were also obtained from GenBank (DQ006233-DQ006267, AY803813-AY803895, AJ880539-AJ880677, AY355755-AY355757, AF205087, AF274929, AF467981, AB029937, AB036396, AB043889, NC002805) and other published sources. Most of the sequences were from four previous studies of the species [16,23,27,40]. We did not sequence many new individuals of *P. nigromaculatus *because a large number of sequences were already available. Four nuclear gene sequences (*POMC *AB360151; *TYR *D12514, AY322363. DQ360045) were also obtained from GenBank.

Eight ranid species, *Pelophylax lessonae, P. saharicus, P. perezi, P. porosus, Babina pleuraden, Rana shuchinae, Staurois latopalmatus, Lithobates catesbeianus*, were selected as outgroup taxa. Most of these species were considered closely related to *P. plancyi *and *P. nigromaculatus *[18,20,21,41]. All their sequences were obtained from GenBank (*POMC *AY819106, AB360150; *TYR *DQ360042, DQ360057, AY322347; *Cyt-b *DQ474177, EU047779, AB029938, AB036402). Different dataset used different outgroup combinations depending on the availability of data. Detailed information of specimens and collecting sites are listed in Additional file 4 and shown in Figure 1.

### Laboratory protocols

We sequenced one fragment from the mitochondrial genome and two fragments from the nuclear genome. The mitochondrial *Cyt-b *gene is one of most frequently used genes in vertebrates for phylogenetic construction, and several previous studies of *P. nigromaculatus *used this gene fragment [27,40]. By using the same fragment, we were able to incorporate the published data in our analysis. From the nuclear genome, we selected the *POMC *and *TYR *as markers because they have been extensively used for ranid species and both demonstrate variability at population level. All primers used in this study are listed in Table 1.

**Table 1:** Primers and annealing temperatures used for PCR and sequencing in this study

Gene	Primer	Primer Sequences (5'-3')	Annealing T	Reference
*Cyt-b*	B104(F)	AAC ATC TCT GCA TGA TGA AAC TTC GG	55°C	This study
	B829(R)	AT TGA GCG AAG GAT GGC GTA GGC GAA		
*POMC*	POMC-1(F)	GAA TGT ATY AAA GMM TGC AAG ATG GWC C	50°C	Wiens et al. [56]
	POMC-2(R)	TAY TGR CCC TTY TTG TGG GCR TT		
	POMC-3(F)	TCT GCM GAR TCW CCY GTG TTT CC	50°C	
	POMC-4(R)	TGG CAT TYT TGA AAA GAG TCA T		
*TYR*	TyrlB (F)	AGG TCC TCY TRA GGA AGG AAT G	50°C	Bossuyt and Milinkovitch [57]
	Tyr1G(R)	TGC TGG GCR TCT CTC CAR TCC CA		

Genomic DNA was isolated from liver or muscle tissues using a standard phenol/chloroform extraction protocol [42]. Standard polymerase chain reaction (PCR) amplification was performed with an annealing temperature that was optimized for each primer pair (Table 1). All PCR products were verified on 1% agarose gels and purified using QIAquick PCR purification kits (Qiagen). The purified products were directly cycle-sequenced with the same primers from both directions. All DNA sequencing reactions were performed using BigDye terminator sequencing chemistry with an ABI 3730 (Applied Biosystems) automatic sequencer.

Nuclear fragments that contained overlapping peaks (double nucleotide calls), were cloned using a pGEM-T Easy Vector System I (Promega) to verify the sequence of each haplotype in heterozygous individuals. Restriction enzyme reaction (EcoRI blue/white cloning qualified EcoRI restriction enzyme, Promega) was first performed using plasmid extraction in white clones to determine the colonies that contain positive inserts. Bacterial-PCR was then performed using selected positive individual clones as templates. The PCR products were purified and sequenced following the same protocols specified above.

### Data analysis

All sequences were checked and edited using BioEdit (version 7) [43]. All alignments were completed using MacClade (version 4) [44]. All three fragments were from coding regions and therefore the alignment was straightforward. Prior to phylogenetic analysis of the haplotypes, recombination tests were conducted for all nuclear gene sequences. If recombination occurs within a fragment, phylogenetic methods that produce cladograms (bifurcating trees) would be inappropriate.

We used Sawyer's [45] method to test for recombination. Following the author's recommendation, the default parameters of the computer program Geneconv (Version 1.81) [46] were used, which include the highest acceptable *P *value of 0.05, Bonferroni correction for multiple comparisons, scanning sequence pairs, and a permutation of 10,000. The mismatch penalties parameter was varied from small (gscale = 1) to infinite (gscale = 0) to allow sequence mismatch within each potentially conversed gene fragment.

A phylogenetic analysis was conducted to establish the genealogy of the DNA haplotypes. Both maximum parsimony method and Bayesian inference were used. The parsimony analysis was conducted with PAUP* (version 4.0b10) [47]. All characters were equally weighted and unordered. All phylogenetically uninformative characters were excluded from analysis. Heuristic searches with 1000 random sequence addition replicates were used with tree bisection reconnection (TBR) branch swapping. Due to the large number of similar haplotypes, a 100,000,000 rearrangement limit was imposed on each replicate to reduce the computation time. Nodal support was estimated with bootstrap analyses [48] using 100 replicates. Within each bootstrap replicate, 10 random sequence addition replicates were conducted with a 100,000,000 rearrangement limit on each replicate.

The Bayesian analysis was conducted with MrBayes (version 3.2) [49]. A best-fit DNA substitution model was first selected by MrModeltest (version 2.1) [50]. A flat "prior" setting was used in MrBayes and four Markov chains were executed. Each dataset was run for 10,000,000 generations and trees were sampled every 500 generations. We used the last 10,000 sampled trees to estimate the consensus tree and the Bayesian posterior probabilities, and all other trees were designated as "burn-in". Tracer (version 1.4) [51] was used to plot the resulting likelihood values and to determine when the Markov chains reached convergence. Two separate runs, which included a total of four independent tree searches, were conducted and the resulting trees were compared and pooled.

For divergence time estimates, a Bayesian method with computer program BEAST (version 1.5.1) [52] was used. We were only interested in the divergence times of the major clades, therefore, a simplified data set was constructed for the BEAST analysis. Most similar haplotypes of *P. fukienensis*, *P. nigromaculatus *and *P. plancyi *were excluded, and only eleven haplotypes were included, which represented the major lineages. Nine additional taxa, *Glandirana rugosa *(AF205093), *Pelophylax bedriagae *(DQ474141), *P. cretensis *(DQ474147), *P. epeirotica *(DQ474153), *P. kurtmuelleri *(DQ474156), *P. lessonae *(EU047779), *P. perezi *(DQ902146), *P. porosus *(AB029938, AB036402), and *P. saharicus *(DQ474177) were introduced to provide a calibration point and a root for the tree. We used the separation of the island of Crete from the mainland as a calibration point, which corresponds to the separation of *P. cretensis *from the common ancestor of *P. bedriagae, P. epeirotica *and *P. kurtmuelleri *[21]. Geological studies dated the separation at 5-5.5 MYA [53,54]. A HKY+I+G was used to describe the substitution model, a Yule process was used to describe speciation and an uncorrelated lognormal (UCLN) model was used to describe the relaxed clock [55]. BEAST was run for 80,000,000 generations with samples taken every 1,000. Three independent MCMC runs were conducted.

## Authors' contributions

All authors participated in the project design and sample collecting. KL and FW carried out most of the fieldwork and molecular data collection, and participated in data analysis. WC, LT and MM commented on manuscript drafts. KB participated in data collecting and analysis. JF conceived the project, participated in data analysis and finalized the manuscript. All authors read and approved the final manuscript.

## Supplementary Material

Additional file 1 **Aligned *cytochrome b *sequence data for *Pelophylax fukienensis*, *P. nigromaculatus *and *P. plancyi***. Click here for file

Additional file 2 **Aligned *POMC *sequence data for *Pelophylax fukienensis*, *P. nigromaculatus *and *P. plancyi***. Click here for file

Additional file 3 **Aligned *tyrosinase *sequence data for *Pelophylax fukienensis*, *P. nigromaculatus *and *P. plancyi***. Click here for file

Additional file 4 **Specimen and sampling site information for *Pelophylax fukienensis, P. nigromaculatus *and *P. plancyi***. Click here for file
