# Supplementary material for: Rampant historical mitochondrial genome introgression between two species of green pond frogs, Pelophylax nigromaculatus and P. plancyi
Source: BMC Evol Biol. 2010 Jun 29;10:201. doi: 10.1186/1471-2148-10-201 (PMC2909235; doi:10.1186/1471-2148-10-201)
Supplement: Additional file 4 — Specimen and sampling site information for Pelophylax fukienensis, P. nigromaculatus and P. plancyi. [file 1471-2148-10-201-S4.DOC]

**Additional file 4**. Specimen and sampling site information for *Pelophylax fukienensis, P. nigromaculatus* and *P. plancyi.* Site numbers in parentheses correspond to site numbers in Figure 1.

*Pelophylax fukienensis* (n=13)

1. (=site 17) Shao Wu Co., Fujian Province, China. 27.37522°N, 117.53284°E. CNU4798, CNU4800-1, CNU5324-6, CNU5342-8 (n=13)

2. (=site 18) Taipei, Taiwan. From GenBank.

*Pelophylax nigromaculatus* (n=60)

1. (=site 2) South Korea. MMS965-7 (n=3)

2. (=site 3) Dan Dong, Liaoning Province, China. 40.12°N, 124.37°E. CNU5405-6, DD01-4 (n=6)

3. (=site 4) Huai Rou, Beijing, China. 40.32°N, 116.62°E. CNU4972-3 (n=2)

4. (=site 4) Ping Gu, Beijing, China. 40.13°N, 117.1°E. PG002-6 (n=5)

5. (=site 20) Xiang Fan, Hubei Province, China. 32.1293°N, 112.1588°E. CNU4955, CNU4957-9, CNU4961 (n=5)

6. (=site 32) Li Chuan, Hubei Province, China. 30.41879°N, 108.78294°E. CNU4916-9, CNU4921, CNU4924-5 (n=7)

7. (=site 21) Yi Chang, Hubei Province, China. 30.9666°N, 111.2613°E. CNU4940-2, CNU4946, CNU4948 (n=5)

8. (=site 6) Luo Shan, Xin Yang, Henan Province, China. 32.20325°N, 114.5644°E. XYLS001 (n=1)

9. (=site 7) Huo Qiu, Anhui Province, China. 32.31861°N, 116.2922°E. HQ001 (n=1)

10. (=site 10) Wu Hu, Anhui Province, China. 31.19694°N, 118.52629°E. CNU5143 (n=1)

11. (=site 9) Huo Shan, Anhui Province, China. 31.42724°N 116.33213°E. CNU5219 (n=1)

12. (=site 11) Xiao Shan, Zhejiang Province, China. 30.1598°N, 120.25879°E. CNU5096 (n=1)

13. (=site 13) Qu Zhou, Zhejiang Province, China. 29.26976°N, 118.91850°E. QLY16-7 (n=2)

14. (=site 15) Jiu Jiang, Jiangxi Province, China. 29.59381°N, 115.87798°E. CNU5268 (n=1)

15. (=site 19) Jing Gang Shan, Jiangxi Province, China. 26.73553°N, 114.30071°E. CNU5291 (n=1)

16. (=site 17) Shao Wu, Fujian Province, China. 27.37522°N, 117.53284°E. CNU4817, CNU4819-21, CNU4839 (n=5)

17. (=site 29) Xing Shan, Hubei Province, China. 31.33488°N, 110.76156°E. XS001 (n=1)

18. (=site 30) Ba Dong, Hubei Province, China. 30.90124°N, 110.34852°E. BD001 (n=1)

19. (=site 31) Jian Shi, Hubei Province, China. 30.61388°N, 109.72865°E. JS002 (n=1)

20. (=site 28) Xuan En, Hubei Province, China. 29.97850°N, 109.49275°E. XE051 (n=1)

21. (=site 24) Li Xian, Hunan Province, China. 29.558°N 112.013°E. LX060 (n=1)

22. (=site 27) Lai Feng, Hubei Province, China. 29.51260°N, 109.41629°E. LF053 (n=1)

23. (=site 26) Zhang Jia Jie, Hunan Province, China. 29.13030°N, 110.44161°E. ZJJ054 (n=1)

24. (=site 34) Zhong Jiang, Sichuan Province, China. 31.0°N, 104.6°E. XM186 (n=1)

25. (=site 36) Mt. Omei, Sichuan Province, China. 29.567°N, 103.417°E. IOZ4022 (n=1)

26. (=site 35) Fu Shun, Sichuan Province, China. 29.2°N, 104.9°E. XM2001 (n=1)

27. (=site 37) Lei Bo, Sichuan Province, China. 28.2°N, 103.5°E. 200019 (n=1)

28. (=site 38) Xi Chang, Sichuan Province, China. 27.9°N, 102.2°E. IOZ3743 (n=1)

29. (=site 39) Mi Yi, Sichuan Province, China. 26.77511°N, 102.11515°E. IOZ3670 (n=1)

## Pelophylax plancyi (n=320)

1. (=site 1) Soweon-ri, Taean-gun, ChungcheongNam-do, South Korea. 36.80392°N, 126.15378°E. MMS541-2 (n=2)

2. (=site 4) Shun Yi, Beijing, China. 40.05262°N, 116.79648°E. CNU5465-74 (n=10)

3. (=site 5) Tai An, Shangdong Province, China. 35.96940°N, 116.36008°E. CNU4974-85 (n=12)

4. (=site 6) Luo Shan, Henan Province, China. 32.18603°N, 114.53368°E. CNU5220-34 (n=15)

5. (=site 8) Liu He, Jiangsu Province, China. 32.28750°N, 118.77539°E. CNU5116-7, CNU5119-31 (n=15)

6. (=site 7) Huo Qiu, Anhui Province, China. 32.34480°N, 116.28205°E. CNU5165-79 (n=15)

7. (=site 10) Wu Hu, Anhui Province, China. 31.19568°N, 118.52387°E. CNU5134-42, CNU5144-9 (n=15)

8. (=site 9) Huo Shan, Anhui Province, China. 31.4062°N, 116.33251°E. CNU5197-211 (n=15)

9. (=site 14) An Qing, Anhui Province, China. 30.86504°N, 117.12958°E. CNU5450-64 (n=15)

10. (=site 12) Huang Shan, Anhui Province, China. 29.72756°N, 118.35274°E. QLY39, QLY87, QLY88, QLY89, QLY94, QLY97, QLY112, CNU5691, CNU5693-7, CNU5699-700, CNU5702-5 (n=19)

11. (=site 11) Xiao Shan, Zhejiang Province, China. 30.16016°N, 120.25818°E. CNU5078-92 (n=15)

12. (=site 20) Xiang Fan, Hubei Province, China. 32.11482°N, 112.13349°E. CNU4850-4, CNU4856-73, CNU4876-8, CNU4880-2 (n=29)

13. (=site 22) Yi Chang, Hubei Province, China. 30.48616°N, 111.84613°E. CNU4884-904 (n=21)

14. (=site 27) Lai Feng Co., Hubei Province, China. 29.50616°N, 109.40987°E. CNU5349-54 (n=6)

15. (=site 33) Guang An Co., Sichuan Province, China. 30.45011°N, 106.60909°E. CNU3048 CNU3073 (n=2)

16. (=site 26) Zhang Jia Jie, Hunan Province, China. 29.11375°N, 110.50511°E. CNU5355-68, CNU5407 (n=15)

17. (=site 24) Chang De, Hunan Province, China. 29.24929°N, 112.01415°E. CNU5391-9, CD01-11 (n=20)

18. (=site 24) Li Quan, Hunan Province, China. 29.30641°N, 112.16194°E. LQ001-15 (n=15)

19. (=site 25) Li Xian, Hunan Province, China. 29.69376°N, 111.66599°E. CNU5369-83 (n=15)

20. (=site 23) Yue Yang, Hunan Province, China. 29.15754°N, 113.11793°E. CNU5430-44 (n=15)

21. (=site 15) Jiu Jiang, Jiangxi Province, China. 29.59441°N, 115.88283°E. CNU5262-7, CNU5269-77 (n=15)

22. (=site 16) Qian Shan, Jiangxi Province, China. 28.32410°N, 117.69696°E. CNU5293-307, (n=15)

23. (=site 17) Shao Wu, Fujian Province, China. 27.37522°N, 117.53284°E. CNU5336-9 (n=4)
